# Exploring the early impacts of drug decriminalization on harm reduction and opioid agonist treatment service operations and delivery in British Columbia: insights from key informant interviews

**DOI:** 10.1186/s12889-025-25479-x

**Published:** 2025-12-07

**Authors:** Cayley Russell, Savannah Torres-Salbach, Laura MacKinnon, Rita Shahin, Dylan Griffith, Kate Hodgson, Charlene Burmeister, Courtney Amoraal, Frank Crichlow, Sameer Imtiaz, Jürgen Rehm, Farihah Ali

**Affiliations:** 1 Institute for Mental Health Policy Research, Centre for Addiction and Mental Health (CAMH), 250 College St, Toronto, ON M5T 1R8 Canada; 2 Ontario Node, Canadian Research Initiative in Substance Matters (CRISM), Rm.906, 250 College St, Toronto, ON M5T 1R8 Canada; 3 Department of Family Practice, University of British Columbia, 5950 University Blvd, Vancouver, BC V6T 1Z3 Canada; 4 Toronto Public Health, 55 John St, Toronto, ON M5V 3C6 Canada; 5 Kootenay Insurrection for Safe Supply, 40-622 Front St, Nelson, BC V1L 4B7 Canada; 6 Department of Nurse Practitioners, Vancouver Coastal Health, 520 West 6th Ave, Vancouver, BC Canada; 7 AVI Community Health Services, 713 Johnson St, Victoria, BC V8W 1M8 Canada; 8 British Columbia Centre for Disease Control, Vancouver, BC V5Z 4R4 Canada; 9 Canadian Society of Addiction Medicine, 53 Sunlake Rd. SE, Calgary, AB T2X 3G8 Canada; 10 Canadian Association of People Who Use Drugs (CAPUD), 68 Highfield Park Dr Suite 102, Dartmouth, NS B3A 1X4 11 Canada; 11 Dalla Lana School of Public Health, University of Toronto, 480 University Ave, Suite 300, Toronto, ON M5G 1V2 Canada; 12 Department of Psychiatry, University of Toronto, 1 King’s College Circle, Toronto, ON M5S 1A8 Canada; 13 Institute of Medical Science, University of Toronto, 1 King’s College Circle, Toronto, ON M5S 1A8 Canada; 14 Campbell Family Mental Health Research Institute, Centre for Addiction and Mental Health (CAMH), 1001 Queen St. West, Toronto, ON M6J 1H4 Canada; 15 Center for Interdisciplinary Addiction Research (ZIS), Department of Psychiatry and Psychotherapy, University Medical Center Hamburg- Eppendorf (UKE), Martinistraße 52, Hamburg, 20246 Germany; 16 Program on Substance Abuse & WHO European Region Collaboration Centre, Public Health Institute of Catalonia, Aragó Street 330, Barcelona, Catalonia 08009 Spain

**Keywords:** Decriminalization, Drug policy, People who use drugs, Harm reduction, Opioid agonist treatment, Service provision: public health

## Abstract

**Background:**

On January 31, 2023, Health Canada granted British Columbia (BC) a three-year (2023–2026) exemption under the Controlled Drugs and Substances Act (CDSA), decriminalizing the personal possession of up to 2.5 g of certain unregulated drugs among adults (18+) without arrest, seizure of drugs, or criminal penalty. A key objective was to increase awareness, engagement, and retention in harm reduction (HR) and opioid agonist treatment (OAT) services by reducing stigma and enhancing service access. In May 2024, however, the policy was amended to re-criminalize drug use and possession in public spaces. This study examines how decriminalization and its subsequent amendment affected HR and OAT service operations from the perspective of service providers across BC.

**Methods:**

Between October 23rd, 2024 and January 29th, 2025, 18 semi-structured virtual key informant interviews were conducted with HR and OAT service providers across BC. The interviews examined participants’ experiences with decriminalization and its amendment, and how these policy changes impacted service operations and delivery. Thematic analysis was used to identify patterns across interview data.

**Results:**

Key informants reported few operational changes following decriminalization, with no major adjustments to service delivery. Despite expectations of increased client engagement, sites received no additional funding and faced ongoing staffing and resource shortages. Informants emphasized that systemic issues—such as the toxic drug supply and rising homelessness—had a greater impact on service use than the policy itself. A lack of clear communication and site-specific training resulted in uncertainty and confusion, further limiting frontline staff’s ability to respond effectively to policy changes.

**Conclusion:**

Key informants perceived that decriminalization did not lead to immediate changes in HR and OAT site operations. Longstanding systemic barriers continued to limit service capacity and policy impact. Providers highlighted the need for sustained investment in housing, staffing, and supervised spaces to support meaningful engagement and reduce stigma. Addressing these foundational issues was seen as essential for realizing the policy’s intended public health objectives.

**Supplementary Information:**

The online version contains supplementary material available at 10.1186/s12889-025-25479-x.

## Introduction

Canada is in the midst of an ongoing drug toxicity and overdose crisis [1]. For years, the province of British Columbia (BC) has consistently reported the highest rates of overdose deaths related to unregulated drug use [2], prompting a public health emergency declaration in 2016 [3]. In response, BC steadily expanded its harm reduction (HR) and opioid agonist treatment (OAT) initiatives [4, 5].

HR and OAT form part of a continuum of care for people who use drugs (PWUD). HR encompasses a broad range of policies, programs, and practices aimed at minimizing the harms associated with drug use, without requiring abstinence [6]. Key HR interventions include supervised consumption sites (SCS) and overdose prevention sites (OPS), which offer safe, monitored spaces for drug consumption [7, 8]. SCS are permanent, federally exempt facilities staffed by trained personnel (e.g., including nurses, physicians, social workers, and peers), offering services such as overdose response, drug checking, wound care, and testing or support for blood-borne viruses and sexually-transmissible infections (BBV/STIs). OPS often provide similar services but are temporary, peer-led programs operating legally through provincial ministerial orders, or in some cases, illegally, to meet urgent community needs. By providing supervised spaces for consumption, harm reduction supplies, and wrap-around services, SCS and OPS reduce fatal and non-fatal overdoses, lower high-risk drug use behaviors associated with BBV/STIs, and help combat stigma [9, 10].

While HR services aim to prevent immediate harms, OAT represents a broader HR approach focused on long-term stabilization. OAT, the gold standard treatment for opioid use disorder (OUD), involves prescribed medications, such as methadone and buprenorphine/naloxone [11, 12]. By reducing withdrawal symptoms and cravings, OAT helps PWUD stabilize their health, lower their risk of relapse and overdose, and improve overall quality of life, aligning with core harm reduction principles [13, 14].

While OAT is often presented as a harm reduction strategy, it is rooted in addiction medicine, a field that has historically implemented disempowering practices. Measures such as witnessed dosing, urine screening, and punitive dose adjustments for non-prescribed substance use can undermine client autonomy and create barriers to care [15]. Structural challenges, including provider stigma and logistical obstacles, further limit access, echoing barriers seen in non-addiction healthcare settings [16–18].

This highlights the importance of integrating HR and OAT services. Effective integration enhances both programs by providing low-barrier, non-judgmental access to a range of services [19]. Many HR programs now offer OAT on-site or facilitate direct referrals and outreach, bridging harm reduction and treatment. Co-located and coordinated HR and OAT services maximizes reach and effectiveness, supporting PWUD at various stages of readiness [19]. Embedding HR principles within OAT programs can reduce stigma, promote person-centered care, and enable shared decision-making, ultimately improving engagement, autonomy, and health outcomes [16, 19, 20].

BC has been a leader in HR initiatives, hosting over 50 SCS/OPS [21], which collectively recorded 90,325 visits in January 2025 [22]. Despite these efforts, overdose rates in BC remain high, driven largely by an increasingly unpredictable drug supply, with fentanyl involved in 82% of drug-related fatalities between 2023 and 2024 [2, 22]. In response, the BC government launched a three-year drug decriminalization pilot (2023–2026), reframing substance use as a public health issue rather than a criminal justice matter [23–25]. Effective January 31, 2023, adults (18+) could possess up to 2.5 g of opioids, methamphetamine, cocaine/crack-cocaine, and MDMA/ecstasy, without criminal charges, though trafficking, importation, exportation and production remained illegal. A key aim was to reduce the stigma and criminalization historically associated with drug use, thereby promoting access to health, HR, and social services. To meet this anticipated service demand, in 2023, the provincial government committed approximately $19 million towards the implementation of decriminalization, with funds allocated to stakeholder engagement, the regional distribution of decriminalization resource cards, and the staffing of decriminalization lead positions and 24 proactive outreach positions across health authorities [26, 27].

Although public opinion initially supported decriminalization [28], growing concern over a perceived increase in visible drug use in public spaces eroded confidence and public and political support for the policy [29, 30]. As a result, in May 2024, Health Canada approved a request by the BC government to amend the policy, which effectively re-criminalized public drug use and possession in all spaces except private residences, SCS and OPS, outpatient addiction programs, and designated shelters [24, 31]. Simultaneously, a new province-wide directive was introduced prohibiting drug use outside of designated areas within hospital and acute care settings with integrated OPS [30, 32]. This directive was reinforced by political rhetoric and media coverage highlighting negative incidents, such as nurses being exposed to patients smoking drugs in hospital treatment rooms [29, 33, 34].

Although the re-criminalization amendment and the subsequent hospital directive carry important implications for HR and OAT sites across BC, promoting access and engagement remains a core objective of the decriminalization policy [25]. These services are central to the policy’s overall success, serving as low-barrier, evidence-based interventions that can reduce drug-related risks and improve client outcomes. However, these are longer-term goals given that policy reforms often require time to effect change across systems and to influence public attitudes, institutional practices, and health outcomes [15, 35, 36].

In this context, assessing preliminary service-level impacts remains valuable, especially considering the policy amendment, to determine whether the policy is on track to achieve its intended goals. Initial survey data from the policy’s first year suggest HR and OAT sites experienced minimal service adjustments post-decriminalization, yet faced significant staffing and resource demands, with limited decriminalization training provided [37, 38]. Understanding operational changes, such as variations in client volume, resource allocation, and staff capacity, is essential for both evaluation efforts and practical service planning and delivery.

To explore these early impacts, we conducted qualitative interviews with HR and OAT service providers across the province who are on the front lines of service delivery. This study expands on initial quantitative findings to provide deeper insight into how the policy is unfolding in practice. In particular, we examine service providers’ perspectives on the extent to which the decriminalization policy is improving access to HR and OAT services and fostering the conditions necessary for meaningful engagement with PWUD.

## Methods

### Study design, recruitment, and eligibility criteria

This study is part of a national evaluation of BC’s drug decriminalization policy, funded by the Canadian Institutes for Health Research (CIHR; grant # FRN-184698), led by the Ontario Node of the Canadian Research Initiative in Substance Matters (CRISM) [39]. Grounded in a pragmatic epistemological stance and theoretical framework [40, 41], the study prioritized real-world applicability and the lived experiences of key informants, enabling findings that both advance understanding of the policy’s impacts and inform implementation.

To assess the effects of decriminalization on HR and OAT site operations, we used a sequential multi-method approach [42]. Phase 1 involved a quantitative survey circulated to HR and OAT site representatives between March and April 2024 (prior to the amendment) to capture changes to site operations and client dynamics post-decriminalization (see Ali et al. [37] and Russell et al. [38] for methodological details and results). Survey participants were recruited through snowball sampling, leveraging online repositories, our study’s working group (including researchers, community health workers, and PWUD), and harm reduction sites and advocacy groups across BC. Eligible HR sites included low-barrier sites (e.g., SCS/OPS clinics, temporary housing with on-site OPS). Eligible OAT sites included standalone OAT clinics and OAT clinics integrated within HR services, Rapid Access Addiction Medicine (RAAM)/Rapid Access Addiction Care (RAAC) clinics, and Mental Health and Substance Use (MHSU) sites. To ensure informed responses, sites were encouraged to designate respondents with direct operational knowledge, though no strict eligibility criteria regarding respondents or roles were imposed. Phase 2 involved follow-up qualitative interviews with individuals who were eligible for Phase 1 and expressed interest in participating in Phase 2. These individuals were contacted via email and re-consented before the interview.

This paper focuses on the results of the Phase 2 qualitative follow-up study, designed to deepen, contextualize, validate, expand, and complement the survey findings. The interview guide was developed in collaboration with our study’s working group and interview questions were directly informed by the survey items (see supplementary files for interview guides). Questions covered parallel topics as the initial survey, such as services offered, clientele volume and characteristics, funding, referral pathways, training, and any changes experienced as a direct result of decriminalization. However, to further contextualize survey findings, broader questions explored the policy’s impact on site operations, staffing, clients, and communities, as well as key informants’ perceptions of the policy’s effectiveness, anticipated long-term effects on service utilization and operations, and recommendations for policy improvements. Since the Phase I was conducted prior to the May 2024 policy amendment, we further leveraged the follow-up interviews as an opportunity to explore key informants’ experiences regarding the unique impacts of re-criminalization.

### Data collection

All interviews were conducted between October 23rd, 2024 and January 29th, 2025 (post-amendment). Each interview began with a brief interviewer-led questionnaire about site characteristics, services offered, and key informants’ roles. Interviews were conducted virtually via WebEx or by phone, based on informants’ preference, and lasted approximately 45 to 60 min. Questionnaire responses on site characteristics were inputted by the interviewer into Research Electronic Data Capture (REDCap) software, a secure web application for building and managing online surveys and databases [43]. Interviews were audio-recorded, transcribed, and quality-checked by a research team member (STS). All key informants received a $50 Amazon e-gift card for their participation.

### Data analysis

All interview transcripts were imported into NVivo (v. 12) for qualitative analysis using an iterative, thematic analysis approach, following Braun and Clarke’s (2006, 2012) framework [44, 45]. Two research team members (STS, CR) familiarized themselves with the data by reviewing notes, memos, recordings, and transcripts, and engaging in team debrief discussions after interviews. A preliminary codebook was developed based on the interview guide, initial insights, and research questions. Prior to coding, the codebook was reviewed, critiqued, and approved by the study’s working group who provided guidance and feedback throughout the study [39]. One team member (STS) coded all transcripts, while a second coder (CR) coded a subset to enhance reliability and consistency. Codes were grouped into broader themes, which were iteratively developed, refined, and contextualized, with key informant quotes used to support key findings. To maintain anonymity, all data were de-identified, including site types, names, or locations, and participant roles. To further enhance rigor and to ensure data saturation, we employed post-interview memo-writing, data triangulation, and member-checking, drawing on the expertise and lived experience of the study’s working group. The team engaged in reflexive discussions to consider how our disciplinary backgrounds and experiences shaped our interpretations, ensuring analyses remained grounded in participants’ accounts.

Questionnaire data on site characteristics were also exported from REDCap into Microsoft Excel, where basic descriptive statistics (i.e., counts, frequencies) were tabulated.

### Ethics

The study was approved by the Centre for Addiction and Mental Health (CAMH) Research Ethics Board (#2023/169).

## Results

A total of 61 HR and OAT sites completed the initial survey (Phase 1), of which 44 (72%) site representatives expressed interest in participating in the Phase 2 follow-up key informant interviews. After removing duplicate responses to ensure only one representative per organization was included, 40 representatives were invited to participate. Of these, 18 (45%) key informants located across BC completed an interview. Despite multiple follow-up attempts, the remaining invitees either did not respond or were unavailable.

### Key informants’ roles

Key informants held various roles, most commonly program or clinical coordinators (*n* = 6/18; 33%) and executive or board leadership (*n* = 3/18; 17%). Other roles included educators or trainers (*n* = 2/18; 11%), clinicians/physicians (*n* = 2/18; 11%), managers (*n* = 2/18; 11%), registered nurses (*n* = 2/18; 11%), and an outreach worker (*n* = 1/18; 6%).

### HR/OAT site characteristics

#### Services offered

Sites offered a broad range of services (see Table 1 for a complete list). Most sites also provided referrals to other health and social supports, such as clinical health services, shelter/housing support, detox and withdrawal management, food or nutritional support, and group/peer programming.

**Table 1:** HR/OAT services offered by sites (*n* = 18)

Service	Total (*N*; %)	
Naloxone distribution	17	94
Harm reduction supply distribution	17	94
Substance use counselling	12	67
Mental health counselling	12	67
Drug checking	10	56
Opioid agonist treatment (OAT)	10	56
Mobile outreach	10	56
Peer support	10	56
Social and family support	10	56
Health education	9	50
Overdose prevention services	8	44
Safe consumption services (injection only)	7	39
Clinical/wound care	7	39
Safer supply prescriptions	6	33
Blood-borne virus/sexually transmitted infection (BBVSTI) testing	5	28
Safe consumption services (injection and inhalation)	4	22
On-site OAT prescription delivery	4	22
Community syringe/paraphernalia pick-up	3	17
Indigenous-specific services	2	11

### Qualitative findings

Key informants shared valuable insights on how both the initial decriminalization policy and its amendment affected service operations. Findings are presented under the following three themes: 1) Maintaining the Status Quo: Minimal Impacts of Decriminalization on Service Operations, which reflects how decriminalization had little direct impact on service operations, but limited funding and ongoing staffing challenges constrained sites from expanding services to meet anticipated increases in demand; 2) Beyond the Policy: Broader Systemic Issues Impacting Service Engagement, which describes how service engagement increased prior to decriminalization, driven largely by systemic challenges such as the toxic drug supply and rising homelessness, which have undermined decriminalization’s goals; and 3) Unprepared and Unsupported: The Impact of Limited Government Guidance on Staff Roles and Service Delivery, which focuses on how a lack of government guidance and training created confusion and uncertainty around staff roles and service delivery, highlighting the need for clear, actionable direction.

#### Maintaining the status quo: minimal impacts of decriminalization on service operations

Key informants reported that HR and OAT sites did not experience significant operational changes as a direct result of the decriminalization policy. This included no notable shifts in funding structures, operating budgets, hours of operation, collaborations, referrals to or from other organizations, or the implementation of new services. Many informants explained that they saw no need to adjust their service delivery models in response to the policy as their sites were intentionally designed to prioritize accessibility and client engagement. They noted that their services were inherently low-barrier and wrap-around, having been designed to address the complex and diverse needs of the PWUD they serve, for example, acting as a *de facto* decriminalization space where clients are allowed to safely carry and use drugs on-site without penalty. Many informants described this sentiment, suggesting:

*“I don’t think the decriminalization policy really changed much of our service delivery in terms of OAT and outreach and things like that […] I think it’s because*,* particularly in the OAT team*,* that’s a very low-barrier team*,* going to clients*,* trying to provide them with what they need*,* bringing them harm reduction supplies and naloxone. You know*,* so it was kind of stuff that we were already doing.”* (Key Informant 5).

Many key informants emphasized that their programs already offered comprehensive supports and services, and as a result, they made a deliberate effort to maintain the quality and scope of care they had always delivered, namely providing a space to safely carry and use unregulated drugs. As such, many perceived no need to modify their service models in response to the policy:

*“[We made no changes to our services and operations post-decriminalization]*,* and that was poignant. We made a point of that. We didn’t have to change anything. We’ve always done what we needed to do for clients*,* right? So nothing changed. We were glad*,* but nothing changed.”* (Key Informant 3).

Despite this, several informants expressed a desire to expand their services, for example, extending hours of operation, reducing wait times and waitlists, and hiring more staff, particularly in recognition of the policy’s intent to enhance engagement with health and social services among PWUD. However, informants noted that no additional funding or resources were allocated to support this anticipated increase in service demand. Many emphasized that their sites were already operating under significant financial constraints and expressed confusion and frustration over the lack of new funding following the policy’s rollout. As a result, sites were unable to hire or train additional qualified staff, expand services or capacity, or extend operating hours:

*“I also think it’s about [the site’s] lack of sustainable funding. It’s also impossible to hire*,* we are really struggling with hiring professionals into the roles […] because we don’t have sustainable funding […] also funding to be able to be open more hours and to offer additional services. We would have loved to have been able to expand*,* and always be able to expand services*,* but it’s about funding.”* (Key Informant 7).

Informants highlighted that their ability to expand or scale up operations was also limited by significant challenges recruiting and retaining qualified staff. In particular, they noted difficulties in securing staff with specialized expertise in addiction, such as nurses and clinical support workers. These challenges were attributed to a combination of systemic factors, such as a limited pool of qualified professionals, unstable and insufficient funding, and high rates of burnout, all of which made it increasingly difficult to maintain adequate staffing levels:

*“We’re having challenges with hiring and maintaining staff for sure. And definitely on the nursing side*,* it has been quite challenging. […] I think there is a high occurrence of burnout and moral distress experienced by health care workers […] when you see what it is that your client needs*,* and you don’t actually have anything that you can do to help them get that need met. So not having the resources that you need to support your clients really wears on folks […] and the loss too. There’s so much loss.”* (Key Informant 9).

#### Beyond the policy: broader systemic issues impacting service engagement

While informants described minimal direct impacts of decriminalization on service operations, many observed a continued increase in client engagement and volume post-decriminalization. However, they emphasized that this upward trend preceded decriminalization and could not be definitively attributed to the policy. As one informant noted, *“The numbers [of clients] are always increasing*,* but that was as always”* (Key Informant 3). Several mentioned that while demand had increased, they did not routinely ask clients what prompted their engagement with services, making it difficult to assess whether the policy directly influenced engagement. As one participant shared:

*“It is really hard to pinpoint*,* we don’t really ask our clients*,* ‘what brought you here?’ We just kind of take them where they are and provide the service so*,* […] it is hard to kind of quantify that and to say yes [the increase in clients is] because of [decriminalization].”* (Key Informant 2).

Given the growing demand, many informants described persistent challenges in meeting this increased need for services. As one explained, “*I would say that we’re not meeting the demand […] we always have to turn people away towards the end of the day because our appointments fill up”* (Key Informant 12). Informants stressed that rising service demand was not necessarily attributable to the policy but likely a reflection of broader systemic factors. For instance, the increasingly toxic and unpredictable drug supply, particularly the increase in xylazine (i.e., ‘tranq dope’) and benzodiazepines, introduced complex and often unfamiliar medical challenges, including severe necrotic wounds and overdoses that were less responsive to naloxone, placing additional strain on service provision. While xylazine emerged around the same time as decriminalization, informants emphasized this was coincidental and part of a broader worsening of structural conditions, not a direct policy effect. One informant shared:

*“Xylazine is a bit more common [since decriminalization]. Tranq is what they call it […] I think that’s the only difference in that time span that I’ve seen. […] the wounds are extensively worse and they don’t heal because they don’t get as much blood flow […] Also*,* the overdoses*,* you can use Narcan for opioids*,* but then benzodiazepines have been available on the street far before this whole [decriminalization]*,* [recriminalization]*,* but I think xylazine came out in that [decriminalization] period.”* (Key Informant 17).

The clinical management of xylazine-related wounds placed an additional burden on sites that were already struggling to meet client demands without sufficient funding for medical staff, such as physicians and nurses. The combination of increasing demand, insufficient resources, and limited clinical capacity meant many sites were required to operate beyond their formal training or scope of practice to meet clients’ needs, as one informant described:

*“Our number one demand is more nursing care*,* right? Both from our frontline staff and from our participants in the service […] I think we need a little bit more clinical oversight and just general medical response*,* which nursing would be great. Roughly 70% of our population is dealing with intractable wounds*,* right? So wound care is a huge one. When we do have our communicable disease nurse on site*,* he spends an enormous amount of time dealing with wound care*,* and that’s not his mandate.”* (Key Informant 1).

Additionally, several key informants observed that the increasingly toxic unregulated drug supply made it difficult to engage and retain clients in OAT. They noted a rise in new and transient clients cycling on and off OAT, attributing this to elevated drug tolerances and unintentional dependencies on multiple substances found in the drug supply. These complexities rendered standard OAT dosing insufficient, leading clients to perceive treatment as ineffective and to disengage from it, ultimately complicating the ability of service providers to meet clients’ needs:

*“[We used to have] much better success rates in retaining people in OAT […] I think it’s partly a coincidental correlation*,* not so much a cause of decriminalization*,* it kind of co-occurred with the huge edition of etizolam [a benzodiazepine] into the supply*,* and then now we’re seeing xylazine*,* tranq*,* and other things and that’s actually making it a lot harder for people to remain engaged in OAT because they are physically dependent on more than one class of substance […] that contamination of the illicit supply has really complicated retention.”* (Key Informant 12).

A few informants also noted that clients might misattribute withdrawal symptoms to inadequate OAT dosing, when they are likely experiencing withdrawal from other substances such as benzodiazepines, further complicating treatment adherence:

*“I’ve definitely seen some folks who maybe don’t realize that they might be experiencing benzo withdrawal and are saying like*,* “Oh my methadone or like whatever just isn’t holding me”*,* like*,* “I still feel dope-sick”*,* and I wonder if part of that is benzo withdrawal […] and folks are perceiving that their OAT isn’t effective.”* (Key Informant 9).

In addition to the toxic drug supply, several key informants highlighted the critical role of housing insecurity in shaping clients’ needs and, ultimately, their care trajectories. They noted a substantial increase in the number of unhoused PWUD in their communities, which had been intensifying for years:

*“The unhoused population that’s using substances*,* that’s been on a continuous upward trajectory for at least five years […] I think there’s far more substance use out in public than there was five years ago*,* and that is the lack of housing for people […] That’s all missing in our community.”* (Key Informant 11).

Key informants explained that the lack of low-barrier and supportive housing options remained a substantial barrier to service engagement. Without a stable place to live, individuals often struggled to attend appointments consistently, store medications safely, or access support. Furthermore, the absence of private and safe spaces to use drugs, especially for inhalation, pushed many PWUD to use in public, heightening visibility and fueling criticism of the policy. Several informants described a shift from injection to inhalation as the primary method of drug consumption, partially driven by the belief among PWUD that inhalation carries a lower overdose risk. Informants suggested this amplified the *perception*, not necessarily the *reality*, of increased public drug use:

*“People now smoke their substances overwhelmingly compared to injection […] that change has had a compounding effect where people’s use more directly impacts those around them. I think there’s a perceived increase of public use*,* and not so much an actual increase in public use*,* because of the change in the route from [intravenous] use to smoking.”* (Key Informant 12).

Key informants stressed that these issues stemmed not from the decriminalization policy itself, but from an absence of the infrastructure needed to support it, stating, *“I feel like there weren’t enough solutions*,* like operational [SCS/OPS] sites*,* like whether it’s an inhalation site*,* or a conducive community area where people could use.”* (Key Informant 16).

As a result, key informants suggested the public increasingly linked decriminalization with rising public drug use. This perception fueled opposition, reinforced negative views, and ultimately led to the amendment that re-criminalized public drug use and possession:

*“Of course [decriminalization] failed. It was set up to fail from the very beginning. You can’t simply decriminalize drugs and public drug use but not open spaces for people to safely do it or for people to go during the day so that they’re not out on the street doing it. If you’re so upset by people using publicly*,* then build spaces for them to go to use. And they didn’t do that.”* (Key Informant 3).

Informants indicated that the lack of investment in places for PWUD to use their drugs not only undermined the policy’s goals of increasing engagement in services, but also reinforced stigma, an issue that they felt had worsened significantly post-decriminalization. Several informants described increased public hostility towards PWUD stating, *“[Stigma has increased] absolutely*,* absolutely tenfold.”* (Key Informant 7), which they believed discouraged individuals from accessing services.

Although decriminalization was intended to reduce stigma and increase service engagement, many informants questioned whether those goals were achievable under the current conditions. With widespread stigma, misinformation, and a lack of structural supports, the policy’s intended outcomes felt increasingly out of reach:

*“I don’t think [decriminalization] will [increase engagement and access to services]. Like again*,* it’s an interesting time*,* it’s a scary time. Currently*,* the loudest voices that we’re hearing right now are the ones full of hate and stigma. […] so I think that it’s an almost impossible time right now for [decriminalization] to be successful.”* (Key Informant 10).

Overall, informants reported rising service demand and care complexity, but attributed these trends to broader structural issues that pre-dated decriminalization. Notably, they emphasized that heightened stigma and insufficient supportive infrastructure left providers overwhelmed and unprepared to support clients, underminining the policy’s rollout.

#### Unprepared and unsupported: the impact of limited government guidance on staff roles and service delivery

Many informants further described a lack of sufficient guidance or training from the provincial government on how to adapt services or orient staff and clients to the initial policy and its subsequent amendment. For instance, one informant remarked that they knew *“No more [about the details of decriminalization] than the average person would have seen in the newspaper”* (Key Informant 13), highlighting a lack of formal communication. Among those who did receive information, most described it as limited, informal, and inconsistent, typically conveyed via mass emails, internal memos, flyers, or word-of-mouth from colleagues. One participant, reflecting on their onboarding experience, noted the absence of structured guidance:

*“I joined the [site] after decriminalization had already been put in place and there was very little information disseminated about [the policy]. […] There was a distinct lack of knowledge on how decriminalization would happen in terms of best practices in our clinic*,* what that would look like. We purely were told*,* ‘Oh*,* hey*,* there’s policies on the intranet*,* go look them up.’”* (Key Informant 12).

Due to a lack of formal and detailed guidance, many felt an unspoken expectation to quickly become knowledgeable about the policy in order to educate and inform clients and support their teams, despite no official requirement to do so. In the absence of structured training, staff were often left to self-educate, spending considerable time trying to understand their clients’ rights under the policy and creating their own educational resources for internal and external use. For some, the burden of independently bridging these knowledge gaps was described as overwhelming. As one key informant explained:

*“The amount of demand that we got from [other organizations] […] to come individually and train everybody on what [decriminalization] meant. […] The demands on us were overwhelming and we felt so unsupported. […] I felt like we didn’t have anywhere near enough resources*,* either human resources*,* or print resources*,* or things that we could share that would reassure people.”* (Key Informant 4).

The lack of guidance and training was particularly concerning in the context of the policy amendment. Several key informants emphasized that there was virtually no communication from the provincial government explaining the goals of the policy reversal or its implications for HR and OAT service delivery. As a result, staff and clients were left in a state of considerable uncertainty, with widespread confusion about what the amendment meant in practice and how services were expected to respond to clients’ queries:

*“There’s a lot of questions from [clients] being like*,* ‘Hey*,* are we still allowed to use? Like*,* what does this mean for us onsite? You know*,* what does it mean for us at home?’ And there was a real lack of knowledge sharing and information.”* (Key Informant 10).

The resulting ambiguity around the policy prompted some sites to revise internal rules in ways that ultimately reduced service accessibility. For instance, a few adopted stricter measures and more rigid protocols, such as temporarily banning clients for ‘splitting or sharing’ drugs onsite, to avoid contravening the amendment or placing clients at risk for arrest for possession under the new regulations. Informants highlighted that these restrictions disrupted service use and, in some cases, pushed clients away from care entirely:

*“When re-criminalization came in May*,* the staff became much more rigid in putting bans in place for people attending the site*,* and it doesn’t really matter for a person if you tell them you’re banned from coming back here for 24 hours*,* they hear the word ‘banned’ and they don’t return […] and would disengage from care entirely where they had previously been attending on a semi or fully regular basis.”* (Key Informant 12).

Around the same time as the re-criminalization amendment, the BC Ministry of Health issued a directive restricting substance use in hospitals and other acute care settings across the province. The directive also expanded the authority of on-site security to intervene, and, in some cases, use force when patients were found using or possessing drugs outside of designated areas (e.g., onsite OPS). Informants noted that this directive disrupted service engagement and compromised patient safety, often escalating into violent incidents:

*"Our security officers […] were given the mandate of preventing anyone from using substances anywhere. And so we’ve seen an increase […] of physical violence both directed towards patients and towards security guards who have been given this impossible task of intervening whenever they suspect somebody of using drugs […]*,* which is a mandate that causes violence every day because you have security guards approaching people every hour saying*,* “Hey*,* do you belong here? Are you supposed to be here? Are you a patient?” […] and so it’s just constant conflict”* (Key Informant 4).

One key informant expressed uncertainty around how to adapt services to comply with the directive, specifically noting the lack of clear guidance on whether drugs should be confiscated, destroyed, or returned, and whether clients had the legal right to retain their personal supply:

*“There’s a lot of misunderstanding of whether security can remove people’s supply from them upon admission to hospital*,* whether people are allowed to continue to carry their substances for personal use when they come to the emergency room*,* and a lot of confusion around what that looks like now.”* (Key Informant 12).

In light of these challenges, key informants emphasized the urgent need for clear, actionable guidance, including training materials that explain the practical implications of both decriminalization and re-criminalization, along with any associated internal or site-specific directives or policies. They expressed a strong desire for consistent, accessible information outlining how evolving policies affect service delivery, noting that such clarity is essential to effectively support clients:

*“It would be helpful to maybe have like a blurb attached of how [the policy is] going to impact each service and how this is relevant to you and your clients*,* if that makes sense. Like if there’s any way that it’s going to impact how we’re going to do things*,* if we could have a heads up.”* (Key Informant 5).

### Discussion

This study provides a timely examination of the preliminary impacts of BC’s decriminalization policy and its subsequent amendment on HR and OAT service operations and delivery across the province. While the policy was intended to reduce stigma and increase service engagement among PWUD, our findings suggest that its implementation and success have been constrained by persistent systemic barriers, inadequate investment in critical resources, and a lack of coordinated, government-endorsed training for service providers. Furthermore, the policy amendment - and the corresponding confusion and uncertainty it caused - may have critically undermined the implementation of decriminalization [46]. This amendment also resulted in operational shifts that reflect more restrictive, enforcement-based approaches which run counter to the spirit of the original policy. As a result, the initial decriminalization policy did not have the necessary infrastructure to facilitate the transformative, system-level improvements that were anticipated, such as enhanced service engagement.

Leading up to and following the implementation of decriminalization in January 2023, the BC government made several notable investments into mental health and substance use services. For example, the province committed $1 million into HR services followed by an additional $215 million in the 2024 budget to expand mental health and addictions care, with $49 million specifically earmarked for HR initiatives [47, 48]. While these investments reflect a broader recognition of the need for enhanced service delivery, they were not explicitly tied to the implementation of decriminalization, and it is not clear how these investments translated into actual service improvements. Informants emphasized that their sites did not receive dedicated financial resources to support expanded service capacity in response to the policy. This disconnect raises questions about how funding was allocated and why these investments have not translated into system-level support for HR and OAT sites.

The province did, however, make several investments specifically related to the policy, including the aforementioned 24 proactive outreach roles meant to facilitate connections to services. These roles have demonstrated success in facilitating access to care for PWUD through dedicated telephone lines, street outreach, and collaborations between service providers, interdisciplinary teams, peers, and law enforcement, making 3,809 connections between August and October 2024 [49]. However, based on informants’ experiences, these investments did not provide significant operational support. For example, most informants described receiving no additional funding specific to decriminalization, raising questions about how health authorities prioritized and distributed these investments. Moreover the implementation of these outreach positions varied across health authorities, with few roles specifically embedded in HR and OAT settings, where they would have substantial impact. The evaluation of the impact of these investments may also be hindered by inconsistent and unclear tracking mechanisms. For example, health authorities submit monthly reports on these connections, such as the number of clients served and connections to external services, but the broad and inconsistently defined metric of ‘connections to care’ may lead to discrepancies in interpretation and reporting, and raises questions about what constitutes a meaningful connection [50]. Early reports from 2023 also highlight challenges operationalizing these indicators, particularly when they are not applicable to the assigned role or the service context (e.g. low-barrier settings that do not track the number of unique clients). These limitations demonstrate the need for a comprehensive data tracking that is co-created with service providers to ensure relevance and applicability across diverse service models. Despite these targeted efforts, key informants emphasized that sites continued to experience overwhelming service demand without corresponding increases in operational support. Persistent staffing shortages and widespread burnout across the sector highlight a potential missed opportunity to bolster the HR and OAT workforce, which is critical to realizing the goals of the policy.

Other initiatives, though promising, have also fallen short of meaningfully alleviating the burden on HR and OAT services. For instance, access to OAT has expanded through the introduction of RAAC/RAAM clinics and the launch of the Opioid Treatment Access Line, which provides same-day virtual prescribing services [21, 51]. Additionally, in 2023, the province introduced a new ‘certified practice - opioid use disorder’ designation, that allows registered and psychiatric nurses to diagnose and treat OUD, including prescribing OAT medications [52]. While this designation is intended to address provider shortages and improve access to care, its uptake and impact have so far been limited. Many HR and OAT sites lack the necessary resources, infrastructure, or clinical supervision to effectively integrate nurse prescribers into care teams [53, 54]. Additionally, high levels of staff burnout and requirements for annual practice and professional development may further hinder the sustainability of these roles [54, 55]. As a result, these initiatives have not yet reduced clinical workloads or alleviated ongoing staffing shortages.

These implementation gaps mirror implementation failures observed in other jurisdictions that have decriminalized unregulated drugs. In Portugal, initial increases in engagement in HR and treatment services among PWUD following decriminalization were later reversed due to a withdrawal of financial investments in the infrastructure required to support the policy, resulting from budget cuts [56]. Moreover, when the U.S. state of Oregon rolled out their decriminalization policy in 2020, it established a state-wide grant program intended to expand access to drug use treatment, HR, and housing services to support the policy’s implementation [57, 58]. However, initial delays in distributing funding undermined service access [59], which eroded political support for the policy and contributed to its rollback in 2024 [60, 61]. Service providers reported struggling to meet increased service demand, citing challenges similar to those identified by our informants, including difficulties hiring and retaining staff [62]. The experiences from these jurisdictions offer a cautionary example for BC. Without early and sustained investment in HR and OAT infrastructure, the long-term goals of decriminalization may not be achieved. This is particularly concerning as the decriminalization pilot is set to conclude in January 2026, with no extension currently in place.

Our findings further suggest that decriminalization was implemented not only amid longstanding structural challenges, such as chronic underfunding and staffing shortages, but also in the context of an increasingly toxic unregulated drug supply [22] and rising rates of homelessness [63]. Informants emphasized that these broader conditions have played a more significant role in shaping service demand and engagement than the policy itself. Provincial surveillance data supports this interpretation, indicating steady increases in the use of HR services both before and after the policy’s implementation [49]. For example, visits to SCS/OPS across BC have maintained their upward momentum since 2021, increasing from 12,436 monthly visits in November 2021 to 81,899 in October 2024, despite the rollback of the decriminalization policy in May 2024 [22, 49]. Similarly, naloxone kit distribution and drug checking service utilization continued to trend upward over the same period.

Informants largely attributed this increased service demand to the heightened toxicity of the unregulated drug supply, primarily driven by the emerging presence of benzodiazepines [64] and xylazine [65] in Canada, which can cause necrotic wounds and lead to overdoses that are not responsive to naloxone [66–68]. Evidence suggests gaps in both service provider and PWUD knowledge and awareness of benzodiazepines and the management of related harms, complicating engagement and care [69, 70]. Further, some informants described how the increased toxicity of the drug supply led to difficulty retaining clients on OAT, reflecting OAT discontinuation trends in BC [71, 72]. These developments underscore the urgent need for investments in medical staffing - particularly nursing - and targeted clinical education to support the detection, clinical management, and coordination of care for benzodiazepine-related cases. While the province’s investment in outreach workers and the certified practice nurse OUD designation reflect recognition of the need for dedicated staffing support, this may also present an opportunity to embed specialized personnel within HR and OAT sites. While the proactive outreach roles funded to support decriminalization primarily emphasize service navigation and law enforcement integration on crisis teams, the province should also invest in expanding the workforce to include key clinical and medical positions—such as nurses, nurse practitioners, and addictions physicians. Enhancing clinical capacity would not only address rising medical complexity but also help stabilize care pathways.

In parallel, rising homelessness has complicated client engagement and continuity of care in the context of decriminalization. Several informants reported an increase in the number of unhoused clients accessing their sites and described difficulties in initiating and retaining these clients in OAT. These trends are reflected in provincial data demonstrating increases in homelessness [63, 73], as well as a gradual decline in OAT prescriptions following the implementation of the policy [22]. Existing literature identifies housing instability as a major barrier to treatment adherence, appointment attendance, and ongoing engagement in care, underscoring the importance of low-barrier, flexible treatment models, such as mobile and shelter-based OAT programs [74, 75]. While these models are essential for engaging people experiencing homelessness, they do not address the underlying structural drivers of housing instability.

The province has acknowledged that decriminalization alone is insufficient to achieve its long-term service engagement goals, recognizing the necessity of investing in housing supports [25]. In 2023–2024, the province launched the Complex Care Housing (CCH) program, a cross-sector initiative designed to provide integrated housing and healthcare services for individuals with concurrent mental health and substance use challenges [76, 77]. Although this program acknowledges the need for robust housing and harm reduction supports, it was not implemented as part of or in direct coordination with the decriminalization policy – much like the government’s broader funding initiatives described earlier [78]. Without better alignment between housing, HR, and health policy, service providers will continue to face major obstacles in supporting clients experiencing homelessness, ultimately limiting the transformative potential of decriminalization.

Importantly, the convergence of increased homelessness, evolving drug use patterns, and insufficient supportive services has intensified stigma and backlash against decriminalization, culminating in the policy’s rollback. Informants emphasized the absence of safe, designated spaces for drug use - particularly for inhalation - as a critical vulnerability in the policy’s framework, since they believe the perceived increase in public drug use was misattributed to decriminalization rather than broader systemic issues. Without stable housing or shelter, PWUD often have no choice but to use drugs publicly, increasing their exposure to stigma and criminalization, even in areas where decriminalization policies are intended to offer protection [79–81]. This shortfall underscores the pressing need for targeted investments into infrastructure to expand SCS and OPS, with a focus on facilities that accommodate inhalation [82]. As of 2024, less than half of BC’s 50 SCS/OPS offered inhalation services, revealing a significant gap [1]. By providing designated, private spaces for drug consumption, these services reduce the visibility of drug use and publicly-discarded drug use equipment [9, 10, 83] - a factor that informants indicated has fueled negative perceptions and community opposition to the policy and PWUD.

The challenges faced during the implementation of BC’s decriminalization policy were compounded by limited training, inconsistent guidance, and unclear communication from the provincial government and regional health authorities. Most informants reported receiving no or only cursory materials, leading to inconsistent practices across sites. This contrasts with early progress reports submitted by the health authorities to the province describing broad efforts to share information, host informational sessions, consult with peer advisors, and develop guidelines, suggesting gaps between planning and frontline HR and OAT service experiences [50, 84, 85]. Portugal’s approach to decriminalization, which embedded drug education into medical and allied health curricula, offers a useful model for preparing the workforce and shifting attitudes toward treating drug use as a health issue [86, 87].

Uncertainty deepened among HR and OAT providers with the 2024 policy amendment and simultaneous hospital directive which prohibited drug use outside designated OPS within acute care settings [32]. Informants expressed concern that the directive granted on-site security personnel the authority to intervene in situations involving aggressive or problematic behaviors and instances of drug use, possession, or dealing. Further, it gave security guards discretion to respond to non-compliance with the policy, including discharging patients or escalating matters to involve police. A few informants reported that this directive led to increased—and at times violent—interactions between PWUD and security or law enforcement, potentially reinforcing stigma and deterring future care-seeking. Notably, the directive failed to specify what should be done with drugs found on patients, creating confusion and inconsistent practices. PWUD have long faced structural barriers, including stigma, discrimination, and, at times, violence, in healthcare settings, creating an unsafe care environment for PWUD and often deterring them from seeking essential services [88–90]. With the first Canadian hospital-based SCS being opened in 2018 [91], integrating HR sites within healthcare settings is an emerging, but promising, practice that offers non-stigmatizing spaces for drug use and support, while acting as a critical entry point into addictions treatment, facilitating connections to community supports upon discharge [92, 93]. While the directive includes plans for staff training to implement the policy in a trauma-informed manner, granting discretionary enforcement powers to security guards may contradict these principles and place PWUD at increased risk of harm [94, 95].

These challenges underscore the inherent difficulty of evaluating decriminalization’s true impact, particularly in a dynamic policy environment shaped by political agendas and multiple overlapping, and oftentimes, conflicting directives. Without clear communication, operational guidance, or mechanisms to systematically track outcomes, it remains difficult to assess the policy’s effects on service delivery or client engagement. Compounding this, the abrupt policy amendment not only reversed key elements of the original initiative, but also compromised the length of time required to meaningfully assess long-term impacts. As a result, disentangling the independent effects of decriminalization from broader structural and operational contexts remains a complex task, limiting the ability to draw robust and definitive conclusions about its influence on service engagement and client outcomes. That said, the limited changes observed may reflect the long-term nature of decriminalization’s goals related to reducing stigma and improving service engagement, as these require sustained, systemic change over many years [15, 36]. While these are long-term goals, our findings, collected 18 months into the policy, suggest minimal observable progress toward achieving them, raising critical considerations regarding the policy’s implementation and the extent to which the necessary structural conditions and training are in place to support its long-term success.

### Strengths and limitations

This study used in-depth qualitative interviews to build on initial survey findings and provide a nuanced understanding of decriminalization’s impacts across HR and OAT sites in BC. However, several limitations must be acknowledged. First, the sample was drawn from a non-representative pool of survey respondents who met specific eligibility criteria and agreed to follow-up interviews, resulting in a convenience sample that may be subject to selection bias. Many eligible providers declined participation due to capacity constraints.

Second, while HR and OAT funding structures and staffing models vary considerably across BC, we did not analyze or report findings by geographical location to protect informant anonymity. This decision limits the specificity and applicability of our findings across regions.

Third, participants’ insights were shaped by their specific roles, levels of involvement, and familiarity with organizational operations, which may not fully capture broader system-level decisions (e.g., budgeting, policy, or staffing), although sites were encouraged to designate study participants with direct operational knowledge. These limitations underscore the importance of triangulating qualitative data with other sources, such as administrative records, to assess the consistency of decriminalization-related efforts across different types of service providers. Additionally, key informants from privately-funded sites also noted that their experiences may differ from those of publicly-funded sites.

Finally, this study reflects provider experiences at a single point in time. Although most participants distinguished between the initial decriminalization policy and the amendment, the rapidly evolving landscape limits conclusions about longer-term impacts. As such, our findings should be interpreted as reflective of the specific context and timeframe in which our data were collected.

## Conclusion

While decriminalization did not produce major operational changes for HR and OAT sites, our findings reveal a growing strain on staff, driven by rising client volumes and increasingly complex needs, exacerbated by rising homelessness and the toxicity of the drug supply. Despite limited direct policy effects, service capacity remains constrained by burnout, resource shortages, inadequate funding, and the lack of standardized training and communication.

To advance decriminalization’s goals, targeted investments are required. Firstly, increased funding and staffing - including clinical expertise - are essential to accommodate rising client demand, manage client care complexity, and reduce workforce burnout. Second, structural barriers must be addressed through supportive infrastructure, including expanded access to housing and SCS/OPS, with particular emphasis on inhalation spaces. Finally, the implementation gaps observed offer important lessons for other jurisdictions pursuing decriminalization. These include the need for early and meaningful consultation with service providers, as well as standardized training and ongoing education. Engaging frontline staff as partners is essential to ensure consistency, foster alignment, and create the conditions necessary for decriminalization to succeed as a health-centered, evidence-informed approach.

## Supplementary Information

Supplementary Material 1.

Supplementary Material 2.

## Data Availability

De-identified data generated or analyzed during this study can be made available upon reasonable request to the corresponding author.

## Abbreviations

BC: British Columbia
BBVSTI: Blood-Borne Virus and Sexually Transmitted Infection
HR: Harm Reduction
MHSU: Mental Health and Substance Use
OAT: Opioid Agonist Treatment
OPS: Overdose Prevention Sites
OUD: Opioid Use Disorder
PWUD: People Who Use Drugs
REDCap: Research Electronic Data Capture
SCS: Supervised Consumption Sites

## References

[1] Government of Canada. Opioid- and Stimulant-Related Harms in Canada Ottawa, Ontario: Government of Canada. 2024 [Available from: Retrieved from https://health-infobase.canada.ca/substance-related-harms/opioids/maps?index=1. Accessed 10 Sep 2021.

[2] B.C. Coroners Service. Unregulated drug deaths - Summary. 2025.

[3] Government BC. Provincial health officer declares public health emergency. Victoria, B.C.; 2016.

[4] Association BCP, Endnote. A History of Opioid Agonist Treatment in B.C. 2021. Available from: https://www.bcpharmacy.ca/tablet/fall-18/endnote-history-opioid-agonist-treatment-bc.

[5] British Columbia Centre for Disease Control (BCCDC). The History of Harm Reduction in British Columbia. 2012.

[6] Hawk M, Coulter RWS, Egan JE, Fisk S, Reuel Friedman M, Tula M, et al. Harm reduction principles for healthcare settings. Harm Reduct J. 2017;14(1):70. 29065896 10.1186/s12954-017-0196-4 PMC5655864

[7] CATIE. Harm reduction in action: Supervised consumption services and overdose prevention sites 2018. Available from: https://www.catie.ca/prevention-in-focus/harm-reduction-in-action-supervised-consumption-services-and-overdose.

[8] Health Canada. Supervised consumption explained: types of sites and services. Ottawa: Government of Canada; 2024.

[9] Kennedy MC, Hayashi K, Milloy MJ, Compton M, Kerr T. Health impacts of a scale-up of supervised injection services in a Canadian setting: an interrupted time series analysis. Addiction. 2022;117(4):986–97. 34854162 10.1111/add.15717 PMC8904318

[10] Dow-Fleisner SJ, Lomness A, Woolgar L. Impact of safe consumption facilities on individual and community outcomes: a scoping review of the past decade of research. Emerging Trends in Drugs, Addictions, and Health. 2022;2:100046.

[11] Canadian Centre on Substance Use and Addiction. Opioid Agonist Therapy. 2024.

[12] Centre for Addiction and Mental Health. Opioid agonist therapy: A synthesis of Canadian guidelines for treating opioid use disorder. Toronto, Ontario: Centre for Addiction and Mental Health (CAMH); 2021.

[13] Santo T, Clark B, Hickman M, Grebely J, Campbell G, Sordo L, et al. Association of opioid agonist treatment with All-Cause mortality and specific causes of death among people with opioid dependence: A systematic review and Meta-analysis. JAMA Psychiatry. 2021;78(9):979–93. 10.1001/jamapsychiatry.2021.0976 PMC8173472 34076676

[14] Degenhardt L, Clark B, Macpherson G, Leppan O, Nielsen S, Zahra E, et al. Buprenorphine versus methadone for the treatment of opioid dependence: a systematic review and meta-analysis of randomised and observational studies. Lancet Psychiatry. 2023;10(6):386–402. 37167985 10.1016/S2215-0366(23)00095-0

[15] Csete J, Kamarulzaman A, Kazatchkine M, Altice F, Balicki M, Buxton J, et al. Public health and international drug policy. Lancet. 2016;387(10026):1427–80. 27021149 10.1016/S0140-6736(16)00619-X PMC5042332

[16] Power N, Archambault L, Perreault M. New challenges and opportunities for opioid agonist treatment access and retention: a scoping review. J Drug Issues. 2024;0:0.

[17] Pijl EM, Alraja A, Duff E, Cooke C, Dash S, Nayak N, et al. Barriers and facilitators to opioid agonist therapy in rural and remote communities in Canada: an integrative review. Subst Abuse Treat Prev Policy. 2022;17(1):62. 36028837 10.1186/s13011-022-00463-5 PMC9412807

[18] Zelenev A, Shea P, Mazhnaya A, Rozanova J, Madden L, Marcus R, et al. Assessment of barrier severity and willingness to enter opioid agonist treatment among people who inject drugs in Ukraine. Drug Alcohol Depend. 2018;190:82–8. 29990648 10.1016/j.drugalcdep.2018.05.027 PMC6292439

[19] Taylor JL, Johnson S, Cruz R, Gray JR, Schiff D, Bagley SM. Integrating harm reduction into outpatient opioid use disorder treatment settings: harm reduction in outpatient addiction treatment. J Gen Intern Med. 2021;36(12):3810–9. 34159545 10.1007/s11606-021-06904-4 PMC8218967

[20] Hall NY, Le L, Abimanyi-Ochom J, Teesson M, Mihalopoulos C. Identifying the most common barriers to opioid agonist treatment in an Australian setting. Aust J Prim Health. 2023;29(5):445–54. 36934460 10.1071/PY22269

[21] B.C. Ministry of mental health and Addictions. Escalated drug-poisoning response actions. Vancouver: BC Gov News; 2024.

[22] B.C. Centre for disease Control. Unregulated drug poisoning emergency Dashboard. October 16, 2023 ed. Vancouver: BCCDC; 2025.

[23] Government of Canada. The Canadian Drugs and Substances Strategy: The Government of Canada’s approach to substance use related harms and the overdose crisis. 2023.

[24] Government of British Columbia. Decriminalizing people who use drugs in B.C. 2024. Available from: https://www2.gov.bc.ca/gov/content/overdose/decriminalization.

[25] B.C. Ministry of Mental Health and Addictions. Decriminalization in BC: S 56(1) Exemption: Request for an exemption to Health Canada from the Controlled Drugs and Substances Act (CDSA) pursuant to Sect. 56(1) to decriminalize personal possession of illicit substances in the Province of British Columbia. Vancouver, BC: British Columbia Ministry of Mental Health and Addictions (MMHA); 2021.

[26] Columbia GoB. Budget and Fiscal Plan 2023/24–2025/26. 2023.

[27] B.C. Ministry of Health. Evaluation of the British Columbia exemption to allow for personal possession of small amounts of illegal drugs. In: (CRISM) ONotCRIoSM, editor. 2025.

[28] Government of Canada. Report: Canadians’ knowledge and attitudes around drug decriminalization: Results from a 2024 public opinion research survey. 2024.

[29] DeRosa K. Nurses speak out about consequences of drug use in hospitals. Vancouver Sun. 2024.

[30] Government of British Columbia. B.C. Moves to ban drug use in public spaces, taking more steps to keep people safe. Vancouver: Government of British Columbia; 2024.

[31] Major D. Ottawa approves B.C.‘s request to recriminalize use of illicit drugs in public spaces. Toronto: CBC News; 2024.

[32] B.C. Ministry of Health. Substance Use and Addictions Management in Hospitals. Vancouver: 2024.

[33] Hodgson K, Lavigne A, Bardwell G. Nurses’ voices used to heighten fears of fentanyl exposure in British Columbian rural hospitals. Can J Emerg Nurs. 2025;48(1).

[34] Matassa-Fung D. BC Nurses’ Union concerned with rise of illicit drugs, weapons inside hospitals. Toronto: Global News; 2024.

[35] Werb D, Rowell G, Guyatt G, Kerr T, Montaner J, Wood E. Effect of drug law enforcement on drug market violence: a systematic review. Int J Drug Policy. 2011;22(2):87–94. 21392957 10.1016/j.drugpo.2011.02.002

[36] Lancaster K, Ritter A, Hughes C. Conceptualising and operationalising stigma in the evaluation of drug policy: a systematic review of the literature. Drug Alcohol Depend. 2015;151:33–44.

[37] Ali F, Russell C, Law J, Imtiaz S, Budau J, Shahin R, et al. Characterizing changes to harm reduction site operations in British Columbia following the implementation of the decriminalization of drugs: findings from a provincial survey. Harm Reduct J. 2025;22(1):122. 40682069 10.1186/s12954-025-01276-y PMC12275259

[38] Russell C, Law J, Hodgson K, MacKinnon L, Shahin R, Crichlow F et al. Examining opioid agonist treatment (OAT) site operations and early signals of change in the first year of British Columbia’s drug decriminalization policy: Insights from a provincial survey. Can J Public Health. 2025;1–3. 10.17269/s41997-025-01060-2 PMC12627284 40489049

[39] Russell C, Ali F, Imtiaz S, Butler A, Greer A, Rehm J et al. The decriminalization of illicit drugs in British columbia: A National evaluation protocol. BMC Public Health. 2024;24(1):2879. 10.1186/s12889-024-20336-9 PMC11490149 39425094

[40] Kaushik V, Walsh CA. Pragmatism as a research paradigm and its implications for social work research. Soc Sci. 2019;8(9):255.

[41] Lushin V, Anastas JW. Harm reduction in substance abuse treatment: pragmatism as an epistemology for social work practice. J Soc Work Pract Addict. 2011;11(1):96–100.

[42] Morse JM. In: Teddlie C, Tashakkori A, editors. Principles of mixed methods and multi-method research design. Thousand Oaks, CA: Sage Publication; 2003.

[43] Harris PA, Taylor R, Minor BL, Elliott V, Fernandez M, O’Neal L, et al. The REDCap consortium: Building an international community of software platform partners. J Biomed Inf. 2019;95:103208. 10.1016/j.jbi.2019.103208 PMC7254481 31078660

[44] Braun V, Clarke V. Using thematic analysis in psychology. Qualitative Res Psychol. 2006;3(2):77–101.

[45] Braun V, Clarke V. Thematic analysis: American Psychological Association; 2012.

[46] Singh Kelsall T, Veark J, Beatrice M. Criminalizing public space through a decriminalization framework: the paradox of British Columbia, Canada. Int J Drug Policy. 2025;136:104688. 39705876 10.1016/j.drugpo.2024.104688

[47] Ministry of Mental Health and Addictions. 2024/25-2026/27 Service Plan. 2024.

[48] Canadian Mental Health Association. BC Budget 2024: Sustained investments in mental health and substance use care, with key opportunities for improvement 2024. Available from: hhttps://bc.cmha.ca/news/bc-budget-2024-mhsu/.

[49] B.C. Ministry of Mental Health and Addictions. Decriminalization Data Report to Health Canada: February 2023-October 2024. 2025.

[50] B.C. Ministry of Public Safety and the Solicitor General. Freedom of Information Request: Progress reports, financial reports or other reports filed by decriminalization project managers from all health authorities. 2023.

[51] B.C. Ministry of mental health and Addictions. New provincewide opioid treatment access line provides same-day access to care. Vancouver: BC Gov News; 2024.

[52] B.C. College of Nurses and Midwives. BCCNM board approves new RN and RPN certified practice for opioid use disorder 2023. Available from: https://www.bccnm.ca/bccnm/Announcements/Pages/Announcement.aspx?AnnouncementID=466.

[53] Atashi V, Movahedi Najafabadi M, Afshari A, Ghafari S. Barriers to effective clinical supervision from the perspective of nurses: a descriptive qualitative study. Nurs Open. 2024;11(1):e2028. 38268257 10.1002/nop2.2028 PMC10721941

[54] Duff E, Fehr C, Shams S, Wintoniw S, Devenney A, Ashfield D et al. A Cross-sectional study of opioid agonist therapy barriers and facilitators. J Nurse Practitioners. 2024;20(4):104914.

[55] Unachukwu IC, Abrams MP, Dolan A, Oyekemi K, Meisel ZF, South EC, et al. The new normal has become a nonstop crisis: a qualitative study of burnout among Philadelphia’s harm reduction and substance use disorder treatment workers during the COVID-19 pandemic. Harm Reduct J. 2023;20(1):32. 36906576 10.1186/s12954-023-00752-7 PMC10008076

[56] Foundation TDP. Drug Decriminilisation in Portugal: Setting the Record Straight. 2021.

[57] Oregon Legislative Assembly. Senate Bill 755. 2021.

[58] Oregon Health Authority. Drug Addiction Treatment and Recovery Act (Measure 110). Available from: https://www.oregon.gov/oha/HSD/AMH/Pages/Measure110.aspx.

[59] Fagan S, Memmott K. Too early to tell: the challenging implementation of measure 110 has increased risks, but the effectiveness of the program has yet to be determined. Oregon Health Authority; 2023.

[60] Oregon governor signs. A bill recriminalizaling drug possession into law. New York City: AP News; 2024.

[61] Naidoo T, turn on drug decriminalisation: International Drug Policy Consortium,. Backpedalling on progress: Understanding Oregon’s U-; 2024 Available from: https://idpc.net/blog/2024/03/backpedaling-on-progress-understanding-oregon-s-u-turn-on-drug-decriminalisation.

[62] Griffin-Valade L, Memmott K. Funding and delivery of measure 110 substance use disorder services shows prgress, but significant risks remain. Oregon Health Authority. 2023.

[63] The Homelessness Services Association of BC, Caspersen J, D’Souza S, Lupick D. 2023 Report on Homeless Counts in B.C.: Homelessness Services Association of BC; 2024.

[64] Canadian Centre on Substance Use and Addiction. CCENDU Bulletin: Risks and harms associated with the nonmedical use of benzodiazepines in the unregulated drug supply in Canada. 2021.

[65] Canadian Centre on Substance Use and Addiction. CCENDU Bulletin: An update on xylazine in the unregulated drug supply: Harms and public health responses in Canada and the United States. 2023.

[66] Zhu DT. Public health impact and harm reduction implications of xylazine-involved overdoses: a narrative review. Harm Reduct J. 2023;20(1):131. 37700329 10.1186/s12954-023-00867-x PMC10498612

[67] Tobias S, Shapiro AM, Wu H, Ti L. Xylazine identified in the unregulated drug supply in British Columbia, Canada. Can J Addict. 2020;11(3)28–32.

[68] D’Orazio J, Nelson L, Perrone J, Wightman R, Haroz R. Xylazine adulteration of the Heroin-Fentanyl drug supply: a narrative review. Ann Intern Med. 2023;176(10):1370–6. 37812779 10.7326/M23-2001

[69] Aronson ID, Ardouin-Guerrier MA, Baus JE, Bennett AS. Barriers to, and facilitators of, checking drugs for adulterants in the era of Fentanyl and xylazine: qualitative study. JMIR Form Res. 2024;8:e56755. 38959505 10.2196/56755 PMC11255526

[70] Hill K, Minahan-Rowley R, Biegacki ET, Heimer R, Sue KL. Providers’ knowledge and perception of xylazine in the unregulated drug supply: a sequential explanatory mixed-methods study. Harm Reduct J. 2024;21(1):148. 39148036 10.1186/s12954-024-01052-4 PMC11328386

[71] Krebs E, Homayra F, Min JE, MacDonald S, Gold L, Carter C, et al. Characterizing opioid agonist treatment discontinuation trends in British Columbia, Canada, 2012–2018. Drug Alcohol Depend. 2021;225:108799. 34087747 10.1016/j.drugalcdep.2021.108799

[72] Ziafat K, Liu L, Kievit B, Papamihali K, Graham B, Otterstatter M, et al. Opioid agonist therapy discontinuation in British columbia: a cross-sectional study of people who access harm reduction services. BMJ Open. 2025;15(1):e090704. 10.1136/bmjopen-2024-090704 PMC11752018 39819909

[73] Dionne M-A, Laporte C, Loeppky J, Miller A. A review of Canadian homelessness data, 2023. 2023.

[74] Gazzola MG, Thompson E, Hoffman K, Saeed G, Baylen C, Madden LM, et al. You just want to kill the pain and get numb: a mixed methods study investigating the lived experiences of individuals experiencing homelessness enrolled in outpatient methadone treatment. J Subst Use Addict Treat. 2025;172:209668. 40057243 10.1016/j.josat.2025.209668

[75] McLaughlin MF, Li R, Carrero ND, Bain PA, Chatterjee A. Opioid use disorder treatment for people experiencing homelessness: a scoping review. Drug Alcohol Depend. 2021;224:108717. 33985863 10.1016/j.drugalcdep.2021.108717 PMC9758007

[76] B.C. Ministry of mental health and addictions. Complex Care Housing. 2024.

[77] B.C. Housing. 2023/24 Annual Service Plan Report. 2024.

[78] Hodgson K, Kerman N, Kalff-Duschenes M, Bardwell G. Room for change: rural low-barrier housing policy and program models for people with substance use and mental health comorbidities. Int J Homelessness. 2024;4(3):181–8.

[79] Aitken C, Moore D, Higgs P, Kelsall J, Kerger M. The impact of a police crackdown on a street drug scene: evidence from the street. Int J Drug Policy. 2002;13(3):193–202.

[80] Ali F, Russell C, Torres-Salbach S, Lo M, Bonn M, Bardwell G, et al. Experiences of stigmatization among people who use drugs in the initial year of British columbia’s drug decriminalization policy: a qualitative study. Int J Drug Policy. 2025;139:104791. 40188704 10.1016/j.drugpo.2025.104791

[81] Debeck K, Wood E, Qi J, Fu E, McArthur D, Montaner J, et al. Socializing in an open drug scene: the relationship between access to private space and drug-related street disorder. Drug Alcohol Depend. 2012;120(1–3):28–34. 21764528 10.1016/j.drugalcdep.2011.06.015 PMC3202661

[82] Bourque S, Pijl EM, Mason E, Manning J, Motz T. Supervised inhalation is an important part of supervised consumption services. Can J Public Health. 2019;110(2):210–5. 30725386 10.17269/s41997-019-00180-w PMC6964381

[83] Yoon GH, Levengood TW, Davoust MJ, Ogden SN, Kral AH, Cahill SR, et al. Implementation and sustainability of safe consumption sites: a qualitative systematic review and thematic synthesis. Harm Reduct J. 2022;19(1):73. 35790994 10.1186/s12954-022-00655-z PMC9255520

[84] Hussey D, Trinder-Widdess Z, Dee C, Bagnall D, Bojangles T, Kesten JM. Co-design of harm reduction materials for people who inject drugs to implement research findings. Harm Reduct J. 2019;16(1):36. 31174536 10.1186/s12954-019-0300-z PMC6555749

[85] Mallakin M, Dery C, Woldemariam Y, Hamilton M, Corace K, Pauly B, et al. From design to action: participatory approach to capacity building needs for local overdose response plans. BMC Public Health. 2023;23(1):774. 37101181 10.1186/s12889-023-15414-3 PMC10132919

[86] Portugal Council of Ministers. Portugal’s National Drug Strategy 1999. 1999.

[87] Drug Policy Alliance. Drug decriminalization in Portugal: Learning from a health and human-centered approach. 2023.

[88] Rehman M, Chapman L, Liu L, Calvert S, Sukhera J. Structural stigma within inpatient care for people who inject drugs: implications for harm reduction. Harm Reduct J. 2024;21(1):53. 38413991 10.1186/s12954-024-00971-6 PMC10900608

[89] Markwick N, McNeil R, Small W, Kerr T. Exploring the public health impacts of private security guards on people who use drugs: a qualitative study. J Urban Health. 2015;92(6):1117–30. 26453195 10.1007/s11524-015-9992-x PMC4675737

[90] Muncan B, Walters SM, Ezell J, Ompad DC. They look at us like junkies: influences of drug use stigma on the healthcare engagement of people who inject drugs in New York City. Harm Reduct J. 2020;17(1):53. 32736624 10.1186/s12954-020-00399-8 PMC7393740

[91] Dong KA, Brouwer J, Johnston C, Hyshka E. Supervised consumption services for acute care hospital patients. CMAJ. 2020;192(18):E476-9. 32366467 10.1503/cmaj.191365 PMC7207181

[92] Pauly B, Wallace B, Pagan F, Phillips J, Wilson M, Hobbs H, et al. Impact of overdose prevention sites during a public health emergency in Victoria, Canada. PLoS ONE. 2020;15(5):e0229208. 32438390 10.1371/journal.pone.0229208 PMC7242015

[93] French R, McFadden R, Lowenstein M, O’Donnell N, Perrone J, Aronowitz S, et al. A qualitative study exploring the feasibility and acceptability of embedding an overdose prevention sites in a U.S. Hospital. J Subst Use Addict Treat. 2025;170:209620. 39805406 10.1016/j.josat.2024.209620

[94] Messinger JC, Suzuki J. Recognizing and reducing the impact of trauma of hospitalization: considerations for persons who use drugs. J Addict Med. 2022;16(1):7–9. 33758115 10.1097/ADM.0000000000000840 PMC8449792

[95] Bailey A, Bishop E, Black AT, Dogherty E, Sedore G, Humchitt M, et al. Creating safe, inclusive spaces for hospital-based health care staff and people who use drugs: an exploratory qualitative study in Vancouver, Canada. Harm Reduct J. 2025;22(1):39. 40114163 10.1186/s12954-025-01158-3 PMC11924779

