# Supplementary material for: Exploring the early impacts of drug decriminalization on harm reduction and opioid agonist treatment service operations and delivery in British Columbia: insights from key informant interviews
Source: BMC Public Health. 2025 Dec 7;26:157. doi: 10.1186/s12889-025-25479-x (PMC12798107; doi:10.1186/s12889-025-25479-x)
Supplement: Supplementary file 2 — Supplementary Material 2. [file 12889_2025_25479_MOESM2_ESM.pdf]

Decriminalization – OAT Site Respondent Interview Guide

Hi, thank you for taking the time to meet with me to conduct the follow-up interview to better understand the impacts of decriminalization on your OAT site's services and operations. Let's take a few minutes to go over the consent form and you can ask any questions you have about the study and ensure you are still interested in participating.

[consent form review]

Okay, great, thank you. Now we can start the interview, which will first start off with a few basic questions, then we will launch into the open ended questions where you can answer as much or as little as you like. If you do not want to answer any questions, please just let us know and we can skip them. Just a reminder that any information you provide will be de-identified and reported in aggregate form so that responses will remain anonymous.

Site & Respondent Information

1. What is the name of your site?

2. What services does your site provide?

   a. Harm reduction supplies distribution (e.g., safer use kits, etc.)
   b. Safe consumption injection services
   c. Safe consumption injection and inhalation services
   d. Overdose prevention services
   e. Drug checking
   f. Safe supply prescriptions
   g. Community syringe/paraphernalia pick-up
   h. Naloxone distribution (including take-home naloxone distribution/training)
   i. Opioid agonist treatment (OAT) prescriptions (including iOAT and TiOAT)
   j. Sexually transmitted and blood-borne infection (STBBI) testing
   k. Mobile outreach
   l. Clinical care/healing (e.g. wound care, vaccinations, primary care)
   m. Substance use counseling
   n. Mental health counseling
   o. Health education
   p. Detox/withdrawal management
   q. Indigenous-specific services (e.g., Elders and/or traditional healers, smudging)
   r. Peer support
   s. Social and family support (shelter/housing, food, drop-in, washing/showering facilities)
   t. Other (please specify): __________________
   u. I don't know

3. Where is your site located (city + health authority)?

4. When did the site open? (e.g., month/year)

5. What is your position at the site?

Okay thank you for answering those questions. Now I'm just going to give a little background information, after which I will ask the open-ended interview questions.

On January 31st, 2023, BC introduced a pilot program decriminalizing the personal possession of 2.5 grams of opioids, cocaine, crack-cocaine, and MDMA. Then, in May 2024, the government re-criminalized the use and possession of drugs in public. This change in policy means that the possession and use of 2.5g of drugs in public spaces is no longer legal and PWUD can only possess or use drugs legally in private (i.e., at a private residence or where people are legally sheltering) or within specific healthcare settings (i.e., overdose prevention, drug checking, or supervised consumption sites, or at places that provide outpatient addiction services).

One of decriminalization's primary goals is to increase PWUD's awareness, access, and engagement with treatment. As such, this interview is a follow-up interview to the survey you previously filled out on your site's behalf examining the impacts of decriminalization. This interview aims to gather more in-depth information on your thoughts and perspectives related to the impacts of decriminalization on your specific OAT site. We are particularly interested in hearing about your experiences working at this site, any changes your site has experienced since decriminalization, and your thoughts on whether or not the policy is reaching this goal. Thank you for participating.

1. We are interested in knowing about the services and operations of your OAT site, and whether these have been impacted by decriminalization.

- Has there been a change in the demand for OAT at your site specifically as a result of decriminalization (e.g., an increase, or decrease)? If so, what was the change, and how has decriminalization influenced the change? What has the impact been? Has there been a change in demand for OAT at your site more generally, not necessarily as a result of decriminalization? If so, what was the change?

- Some of the preliminary data coming out of our evaluation are suggesting that over the last few years (prior to and since decriminalization), there has been a decreasing trend in the number of people engaging in OAT overall across the province. Does this match your experience at your site?

    - If yes, why do you think that may be the case (e.g., tolerance levels not commensurate with dosing, polysubstance (methamphetamine) use? barriers to OAT engagement, etc.)? Do you feel like the co-prescription of safe supply is influencing/contributing to this? Do you feel that decriminalization has had a specific impact on this? If yes/no, how come?

    - If no, why do you think other sites may be experiencing a decrease in engagement?
- Are there additional services your site currently does not offer but you feel are needed specifically in response to decriminalization? If so, what services do you think should be offered, and why? Are there services your site needs more broadly (not related to decriminalization)? If so, what services should be offered and why?
- Have there been any changes in how your OAT site operates specifically as a result of decriminalization? (e.g., changes to hours or days of operation, changes to the infrastructure, offering different/adjunct services, offering additional services, scaling back services, etc.)? If so, which services have changed or been introduced? In which ways have they changed and why (i.e., how has decriminalization specifically influenced the changes in services offered)? How have the changes impacted your site's ability to meet your client's needs?
    - Are there services your site currently does not offer but should because of/in the context of decriminalization? If so, what services do you think should be offered, and why?
    - Are there any changes that you think should be made to better support decriminalization's goal to increase PWUD engagement with health and OAT services? If so, what should be changed, and why do you feel that way?
- Has your site's approach to service delivery been affected by the re-criminalization of drug possession/use in public spaces? If so, in what ways? How has this impacted your operations and the support you provide to clients?
- Have there been any changes to the medications that your site currently offers that have specifically occurred due to decriminalization (e.g., different/new medications)? If so, what was the change, and what prompted it? If so, what has the impact of those changes been?

Have there been any changes to the provision of take-home OAT doses at your site specifically due to decriminalization (e.g., increases or decreases)? If so, what was the change (e.g., following new guidelines, prescribing more/less take-homes, providing take-homes for some medications but not others, providing easier/quicker access to take-homes, changes to who is eligible to receive take-homes?) and what prompted it? If so, what has the impact of those changes been?2. We are interested in knowing about your OAT site's clientele and whether it has been impacted by decriminalization.

- Have you noticed any shifts in the demographics (e.g., age range, gender, ethnicity, housing status, etc.) of your site's clientele since decriminalization? If so, how has it changed? What do you think has driven this change? Have you noticed any shifts in demographics since the re-criminalization of use and possession in public was enacted?
- Has the overall number of clients accessing your services changed since decriminalization? If yes, can you describe the change and why you think the change occurred (e.g., increased capacity, relaxed eligibility requirements, less stigma)? If clientele increased, is this change due to seeing more new clients, or better retention of existing clients, or both? If your site did not experience a change in clientele since decriminalization, why do you think that is the case?
- Does your site have a waitlist for clients to access OAT? If so, what is the average wait time? Has this changed at all since decriminalization? If so, in which ways?
- Has your site become any more known/familiar to clients since decriminalization? If so, why do you think that is the case? Has there been any specific knowledge/marketing campaigns or advertising of your site and its services? What has this looked like? What has been the impact of this? Was decriminalization specifically mentioned in advertising?
    - Does your site actively communicate decriminalization details to clients (e.g., share resources, asked staff to engage with clients on this topic)? If so, how often does this occur? How are these communications delivered/received? If not, do you feel as though your clients would benefit from learning the specifics of decriminalization from your site?
- Have there been any major challenges or barriers that make it difficult for clients to access or benefit from receiving OAT at your site (e.g., location, hours of operation, stigma, police presence, clinical delivery/treatment options/processes) since decriminalization? If so, what are these challenges? Did decriminalization contribute to them? If so, how?
- Have there been any major challenges or barriers that make it difficult for clients to access or benefit from receiving OAT at your site since the re-criminalization of use and possession in public places?

- Have you noticed any changes in police presence around your site since decriminalization? If so, what changes have you observed, and have the changes impacted your clientele's willingness to access your site? If so, please describe.
- Have you noticed any changes in police presence around your site since the re-criminalization of use and possession in public spaces? If so, what changes have you observed? Have the changes impacted your clientele's willingness to access your site? If so, please describe.
    - Have any clients reported fear of police or legal repercussions when accessing services? (e.g., more PWUD using at or around the site to consume drugs in order to avoid criminalization? More/less clients willing to access your site? More/less clients accessing/engaging with the services at your site)?
- Have you noticed any changes in client health outcomes (e.g., overdose rates, mental health, overall well-being) since decriminalization? If so, what have you observed? How has decriminalization contributed to these changes?

3. We are interested in knowing about your OAT site's current staffing and resources and whether these have been impacted by decriminalization.

- Has your specific role or workload changed since decriminalization? If so, in which ways has your role or the workload changed? (e.g., greater/lesser workload, different requirements, changing clientele/needs, changes to staffing/resources)? Has this affected your ability to serve your clients? In which ways? What would you need in order to better support and meet your client's needs?
- Does your site currently have enough staff and resources to meet your site's demand? If not, what would your site need in order to better support/meet your client's needs?

4. We are interested in knowing about your OAT site's funding source and structure and whether these have been impacted by decriminalization.

- What is your site's funding structure? (e.g., non-profit, government-funded, private/public). How is the site primarily funded? Has this changed since decriminalization? If so, how has decriminalization contributed to these changes?
- Has your site's annual budget changed since decriminalization? If so, in which ways (e.g., an increase or decrease)? If so, how has decriminalization contributed to these changes? If so, is the revised budget sufficient to meet your site's demand?

- Has the client payment structure changed since decriminalization (e.g., do you charge clients a fee for service, and do clients have to pay any more/less out of pocket for medications)? If so, what was the change, and what has the impact been?

5. We are interested in knowing about your OAT site’s referral pathways and collaborations with other community services, and whether these have been impacted by decriminalization.

- Has decriminalization influenced your site’s partnerships or collaborations with other health or social services (e.g. housing services, mental health support)? If so, which services? In which ways has decriminalization influenced collaborations/partnerships?
- Have you noticed any changes in the referral process or volume of referrals since decriminalization (e.g., more/less referrals, referrals to different types of support, etc.)? If so, what have the changes been? How has decriminalization influenced these changes?
    - If receiving more referrals, who are you seeing more referrals from? Is this related to decriminalization? Why do you think this may be the case? If sending more referrals, which types of services are you referring clients to more often? Why is this the case? Is this related to decriminalization? If less referrals, why do you think you are referring/being referred less clients? Is this related to decriminalization?
- Have clients mentioned the decriminalization resource cards handed out by police as a reason for accessing your site? If so, how often does this occur?
- Are police referring clients to your site? If so, how is this being done (e.g., verbally providing information, physically escorting clients to the site, by providing resource cards with site information)?
    - Do you think that police distributing resource cards will help or hinder decriminalization’s goal of increasing PWUD engagement in treatment? Why do you feel this way?
- Have your clients mentioned the re-criminalization of public use and possession as a reason for accessing your services? If so, how often does this occur?
- Have any of your clients discussed the decriminalization policy more broadly as a reason for seeking your site’s services? If so, what do they say is the main reason for seeking your site’s services?

6. We are interested in knowing about your OAT site’s decriminalization training.

- Did anyone at your site receive information about decriminalization? Is so, from who? What did the information contain? Who received it? What did they do with it?
    - Did anyone at your site receive decriminalization training? If so, who delivered/offered the training? Who was trained? What was the format/content of the training?
    - If so, were there any knowledge gaps in the training? Please describe the gaps. Are there any improvements that could be made to the training (e.g., format, content, length, etc.) that you would like to suggest?
    - Did anyone at your site receive re-criminalization training (e.g., training regarding use and possession of drugs in public spaces, types of facilities that are exempt from the re-criminalization policy, etc.)? If so, who delivered/offered the training? Who was trained? What was the format/content of the training?
- If your site did not receive training, do you feel as though your staff could benefit from decriminalization training? If so, what impact do you think the decriminalization training would have on your staff? What should this training look like? Are there any specific aspects of decriminalization or re-criminalization you think would be particularly beneficial for your staff to learn about?

7. Do you have any final thoughts or general suggestions on the impacts of decriminalization on your OAT site?

- Are there any other changes at your site due to decriminalization that we haven't discussed?
- Are there any strategies or measures that can be implemented to better achieve decriminalization's goal of increasing treatment engagement and retention?
- In your opinion, do you foresee the decriminalization policy successfully meeting its goal of increasing PWUD' engagement and retention in treatment in the long term? Why or why not?

Thank you for participating.
